# Prevalence of atrial fibrillation: The Swiss population-based CoLaus|PsyCoLaus study

**DOI:** 10.1007/s00059-021-05090-7

**Published:** 2021-12-13

**Authors:** Daryoush Samim, Damien Choffat, Peter Vollenweider, Gérard Waeber, Pedro Marques-Vidal, Marie Méan

**Affiliations:** Division of Internal Medicine, Department of Medicine, Lausanne University Hospital, Rue du Bugnon 46, 1011 Lausanne, Switzerland

**Keywords:** Atrial fibrillation, Prevalence, Electrocardiogram, Epidemiology, Switzerland, Vorhofflimmern, Häufigkeit, Elektrokardiogramm, Epidemiologie, Schweiz

## Abstract

**Background:**

Atrial fibrillation (AF) is the most common arrhythmia worldwide and is associated with increased morbi-mortality. The prevalence of AF in the Western world is increasing; however, reports on the prevalence of AF in the past decade are scarce, and whether the prevalence of AF increased during the last decade in Switzerland remains uncertain. Therefore, using data from a Swiss population-based sample, we aimed to assess the point prevalence of AF from 2014 to 2017 and to investigate determinants of AF.

**Methods:**

A cross-sectional analysis of 4616 Caucasian participants aged 45–86 years (55% women) from a population-based sample was designed to explore the point prevalence and determinants of cardiovascular risk factors in the population of Lausanne, Switzerland. AF was assessed using electrocardiography (ECG) between 2014 and 2017.

**Results:**

Overall, the point prevalence of AF was 0.9% (95% confidence interval [95% CI]: 0.7–1.2%) and the combined AF + atrial flutter (AFL) point prevalence was 1.1% (95% CI: 8.4–1.5%). The point prevalence of AF was higher among men (81% vs. 19% in women) and increased with age, reaching 3.1% in participants aged ≥ 80. In multivariable analysis, male gender (odds ratio and 95% CI: 4.98 [1.01–24.6]) and increasing age (2.86 [1.40–5.87] per decade) were associated with AF.

**Conclusion:**

The point prevalence of AF and of AF + AFL, assessed between 2014 and 2017 in the city of Lausanne (Switzerland), was low but increased with age and in men.

**Supplementary Information:**

The online version of this article (10.1007/s00059-021-05090-7) contains supplementary material, which is available to authorized users.

## Abstract (de)

**Hintergrund:**

Vorhofflimmern (VF) stellt weltweit die häufigste Arrhythmie dar und geht mit einer erhöhten Morbidität und Mortalität einher. In der westlichen Welt nimmt die Prävalenz des VF immer weiter zu, jedoch sind Publikationen zur Prävalenz des VF im vergangenen Jahrzehnt rar, und ob die Prävalenz des VF im letzten Jahrzehnt in der Schweiz gestiegen ist, bleibt unklar. Daher war es das Ziel der Autor*innen, anhand der Daten einer Stichprobe aus der Schweizer Bevölkerung die Punktprävalenz des VF von 2014 bis 2017 zu ermitteln und Einflussfaktoren auf das VF zu untersuchen.

**Methoden:**

Eine Querschnittsanalyse von 4616 kaukasischen Teilnehmer*innen im Alter von 45–86 Jahren (55 % Frauen) aus einer bevölkerungsbasierten Stichprobe sollte dazu dienen, die Punktprävalenz und Einflussfaktoren hinsichtlich kardiovaskulärer Risikofaktoren in der Bevölkerung von Lausanne (Schweiz) zu ermitteln. Das Vorliegen eines VF wurde mittels Elektrokardiographie (EKG) zwischen 2014 und 2017 diagnostiziert.

**Ergebnisse:**

Dabei lag die Punktprävalenz für VF bei 0,9 % (95 %-Konfidenzintervall [95 %-KI]: 0,7–1,2 %), und die Punktprävalenz für die Kombination aus VF und Vorhofflattern betrug 1,1 % (95 %-KI: 8,4–1,5 %). Bei Männern war die Punktprävalenz für VF höher als bei Frauen (81 vs. 19 %), sie stieg mit dem Alter und erreichte 3,1 % bei Teilnehmer*innen im Alter von ≥ 80 Jahren. In der multivariablen Analyse waren männliches Geschlecht (Odds Ratio und 95 %-KI: 4,98 [1,01–24,6]) und erhöhtes Alter (2,86 [1,40–5,87] pro Jahrzehnt) mit VF verknüpft.

**Schlussfolgerung:**

Die Punktprävalenz für VF und für die Kombination aus VF und Vorhofflattern wurde zwischen 2014 und 2017 in der Stadt Lausanne (Schweiz) untersucht und stellte sich als niedrig heraus, sie nahm jedoch mit dem Alter zu und war bei Männern erhöht.

Atrial fibrillation (AF) is the most common arrhythmia worldwide and is associated with increased mortality [1] and morbidity, particularly stroke [2], myocardial infarction, and heart failure [3].

Several studies performed in Western countries showed variable prevalence of AF, ranging from 0.3% to 7.2%, notably because of heterogeneity in their design and in the age groups studied [4–8]. Indeed, the biggest risk factor for AF is age. The prevalence of AF in adults younger than 55 years is very low and increases to 9% in adults older than 80 years [9]. Due to the aging of the population, prediction models show that more than 18 million AF cases can be expected by 2060 in Europe, which correspond to an increase of 200% in 40 years [9–12].

In the UK, based on data from NHS general practitioners, the prevalence of AF rose from 1.71% in 2015 to 2.05% in 2019 [13]. In the Framingham Heart Study, age-adjusted prevalence of AF over periods of 50 years doubled in men and increased slightly in women (12.6 during 1958–1967 to 25.7 per 1000 person-years during 1998–2007 in men, trend *p* = 0.0007; 8.1 to 11.8 per 1000 person-years in women, trend *p* = 0.009). A population-based survey of individuals aged 35–74 years living in the city of Geneva (Switzerland) at the beginning of 2000 showed an overall prevalence of AF below 1% [8].

However, reports on the prevalence of AF in the past decade are scarce, and whether the prevalence of AF increased during the past decade in Switzerland remains uncertain. Therefore, we aimed to assess, using data from a Swiss population-based sample, aged 45–86 years, and ECG data collected between 2014 and 2017, the point prevalence of AF and to investigate determinants of AF and atrial flutter (AFL).

## Methods

### Recruitment of participants

A detailed description of the recruitment procedure for the CoLaus|PsyCoLaus study and the follow-up process has been published [14]. Briefly, the CoLaus|PsyCoLaus study is a population-based cohort exploring the prevalence and determinants of cardiovascular risk factors in the population of Lausanne, Switzerland. Between 2003 and 2006, a nonstratified, representative sample was recruited based on the following inclusion criteria: (a) age 35–75 years, (b) willingness to participate, and (c) Caucasian origin. The first follow-up was performed between April 2009 and September 2012, 5.6 years on average after baseline; the second follow-up was performed between May 2014 and July 2017, 10.9 years on average after baseline. All study periods included an interview, a physical examination, and blood analysis. At the second follow-up, 12-lead electrocardiograms (ECG) were performed. Hence, this study was conducted using data from the second follow-up.

### Electrocardiographic examination and atrial fibrillation assessment

A resting 12-lead body surface ECG was performed using a portable ECG machine (CARDIOVIT MS-2015, Schiller Reomed® AG, Dietikon, Switzerland) with the patients in supine position, at a paper speed of 25 mm/s. Data on basic rhythm, ventricular rate, P waves, PQ interval, QRS width, and QT interval were collected. Atrial fibrillation was defined as irregular RR intervals and no discernible, distinct P waves. Typical AFL was defined as an atrial activity seen as a “saw tooth” pattern (also called “F waves”), especially in the inferior leads (II, III, aVF), with a variable ventricular rate (ratio of atrial-to-ventricular contraction ranging from 4:1 to 2:1). Atypical AFL was defined as an atrial rate of more than 180/min with no F waves in the inferior leads. All ECGs were interpreted by two physicians (DS, FB). In the case of disagreement, a senior cardiologist (JS) was consulted and his interpretation was retained.

For this study, we considered two main outcomes: “pure” AF (noted as “AF” in this study) and combined AF and typical AFL (noted as „AF + AFL“ in this study). Previous studies [8] grouped AF and typical AFL into the same category, as both arrhythmias share the same risk factors and complications [15].

### Covariates

Covariates were collected by self-administered questionnaires, interview, or physical examination. The detailed definitions of the covariates have been described elsewhere [16].

We collected personal and family history of cardiovascular events. Alcohol consumption was self-reported and categorized as alcohol drinkers (yes/no). Smoking status was defined as never, former, and current. Physical activity (PA) was assessed using a validated wrist-worn triaxial accelerometer (GENEActiv, Activinsights Ltd., UK) and classified in accordance with the World Health Organization (WHO) criteria [17].

Overweight was defined as a body mass index (BMI) of ≥ 25 and < 30 kg/m^2^, and obesity as BMI ≥ 30 kg/m^2^. Hypertension was defined as a systolic blood pressure (BP) of ≥ 140 mm Hg and/or diastolic BP ≥ 90 mm Hg and/or presence of an antihypertensive drug treatment.

Blood samples after an overnight fast included levels of total cholesterol, high-density lipoprotein (HDL)-cholesterol, triglycerides, glucose and glycated hemoglobin (HbA_1_c), creatinine, high-sensitive C‑reactive protein (hs-CRP) and pro-brain natriuretic peptide (pro-BNP). Low-density lipoprotein (LDL)-cholesterol was calculated using the Friedewald formula. Dyslipidemia was defined as an HDL-cholesterol < 1 mmol/L in men and < 1.29 mmol/L in women and/or LDL-cholesterol ≥ 4.1 mmol/L (≥ 2.6 mmol/L if personal history of cardiovascular disease [CVD] or diabetes) and/or triglyceride ≥ 2.2 mmol/L, and/or presence of a hypolipidemic drug treatment. Diabetes was defined as a fasting plasma glucose level of ≥ 7 mmol/L and/or a HbA1c ≥ 48 mmol/mol 6.5% and/or presence of oral hypoglycemic or insulin treatment.

Obstructive sleep apnea (OSA) is a risk factors for AF or AFL [12]; thus, we performed a subgroup analysis using participants with OSA data (*n* = 1920), i.e., the Berlin Questionnaire [18]. This instrument was developed to identify people at high risk of OSA in the ambulatory setting. It is composed of three categories, based on different symptoms of sleep or sleepiness. High risk of OSA is defined as ≥ 2 positive categories.

### Exclusion criteria

We excluded patients (a) without ECG data and (b) with ≥ 1 missing covariate(s) mentioned above.

### Statistical analysis

Statistical analyses were performed using Stata version 15.0 for windows (Stata Corp, College Station, TX, USA). Descriptive results are expressed as number of participants (percentage) for categorical variables and as average ± standard deviation or median and [interquartile range] for continuous variables. Bivariate analyses were performed using chi-square or Fisher’s exact test for qualitative variables and Student’s *t *test, analysis of variance, or the Kruskal–Wallis test for quantitative variables. Multivariable analysis was performed using logistic regression and the results are expressed as odds ratio (OR) and 95% confidence interval (CI). Statistical significance was assessed for a two-sided test with *p* < 0.05.

Due to its paroxysmal nature, it was possible that some participants already presented with AF or AFL in the past. As it was unknown who had already presented with these conditions, we hypothesized that participants taking anticoagulants might have AF or AFL. Therefore, we decided to exclude them from the sensitivity analysis.

### Ethical statement

The institutional Ethics Committee of the University of Lausanne, which later became the *Commission cantonale d’éthique de la recherche sur l’être humain* (www.cer-vd.ch) approved the baseline CoLaus|PsyCoLaus study (reference 16/03) and the approval was renewed for the first (reference 33/09) and second (reference 26/14) follow-ups. The study was performed in agreement with the Helsinki Declaration and all participants gave their signed informed consent before joining the study.

## Results

### Characteristics of the sample

**Fig. 1:**
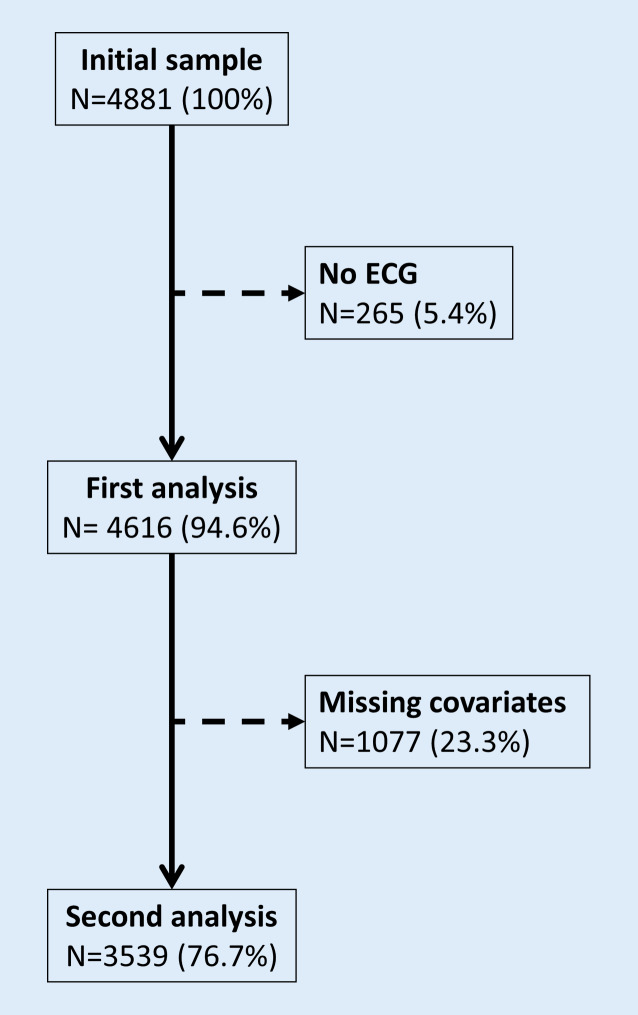
Number of participants excluded and retained for analysis

On the initial 4881 participants, 4616 (94.6%) were retained for analysis. The exclusion criteria are indicated in Fig. 1 and the characteristics of the included and excluded participants are summarized in Supplementary Table 1. Included participants were younger, had lower levels of hypertension, diabetes, dyslipidemia, creatinine, hs-CRP, and pro-BNP, and less frequently reported a personal history of CVD. Included participants also reported higher levels of alcohol units consumed per week and were more physically active. Similar findings were obtained when exclusion criteria were extended to participants with at least one missing covariate(s) (Supplementary Table 2), except that included participants were more frequently overweight and had dyslipidemia and less frequently alcohol drinkers. To summarize, excluded patients were older and sicker, which might explain the absence of ECG data as those patients are harder to keep in a prospective study [19, 20].

### Point prevalence of AF/AFL

**Fig. 2:**
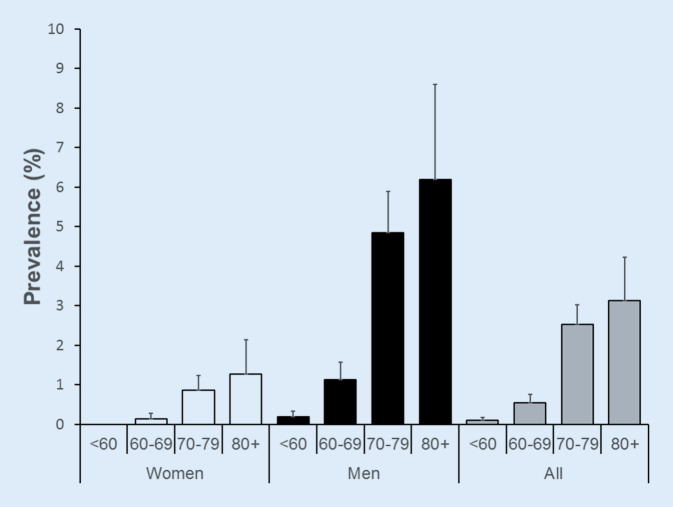
Prevalence of AF according to gender and age categories at second follow-up, CoLaus|PsyCoLaus study, Lausanne, Switzerland

Overall, 42 cases of AF were detected, resulting in an AF point prevalence of 0.9% (95% CI: 0.7–1.2). The point prevalence of AF by sex and age is presented in Fig. 2.

**Fig. 3:**
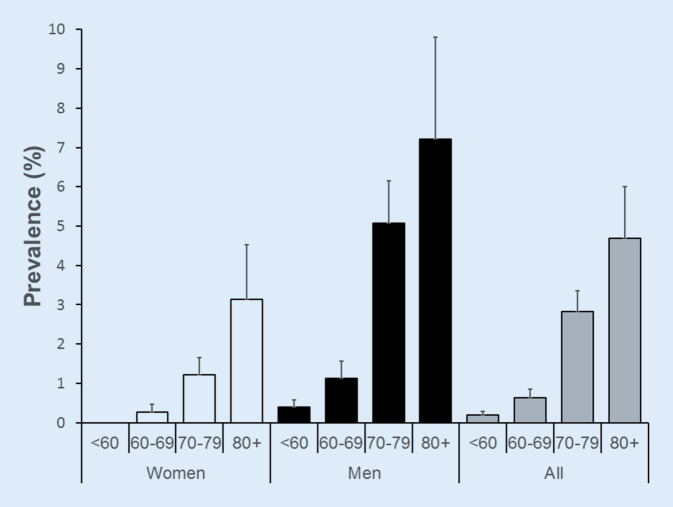
Prevalence of AF + AFL according to gender and age categories at second follow-up, CoLaus|PsyCoLaus study, Lausanne, Switzerland

Furthermore, 10 cases of typical AFL were recorded, but no atypical AFL was detected. Therefore, the combined point prevalence of AF + AFL is 1.1% (95% CI: 0.4–1.5%). The point prevalence of combined AF + AFL by sex and age is presented in Fig. 3.

### Factors associated with AF/AFL

The bivariate comparisons of the clinical and sociodemographic characteristics between AF and non-AF cases are summarized in Table 1. Participants with AF were older, more often men (81%), presented more often with hypertension, dyslipidemia, or diabetes, had higher levels of BMI, and had more frequently a personal history of CVD. Participants with AF also had higher levels of creatinine, hs-CRP, and pro-BNP, were more often alcohol drinkers, reported higher alcohol consumption, and were less physically active. The AF participants were slightly more at risk of OSA but without reaching a statistically significant difference.

**Table 1:** Characteristics of participants with and without AF at second follow-up, CoLaus|PsyCoLaus study, Lausanne, Switzerland

	Non-AF cases	AF cases	*p*
*N*	4574	42	
*Age (years)*	62 ± 10	74 ± 7	< 0.001
*Women (%)*	2531 (55.3)	8 (19.0)	< 0.001
*History of cardiovascular disease (%)*			
Personal	236 (5.2)	12 (28.6)	< 0.001
Family	2262 (49.5)	19 (45.2)	0.586
*Hypertension (%)*	2069 (46.6)	37 (88.1)	< 0.001
*Systolic blood pressure (mm* *Hg)*	127 ± 18	132 ± 25	0.037
*Diastolic blood pressure (mm* *Hg)*	77 ± 11	81 ± 14	0.034
*Body mass index (kg/m*^*2*^*)*	26.4 ± 4.7	29.1 ± 4.4	< 0.001
*Body mass index categories (%)*			0.001
Normal + underweight	1801 (41.7)	7 (16.7)	–
Overweight	1719 (39.8)	19 (45.2)	–
Obese	802 (18.6)	16 (38.1)	–
*Dyslipidemia (%)*	2048 (44.8)	26 (61.9)	0.026
*Diabetes using FPG (%)*	439 (10.1)	11 (26.2)	0.001
*Diabetes using HbA*_*1*_*c (%)*	423 (9.8)	12 (28.6)	< 0.001
*Creatinine (μmol/L)*	79 ± 21	94 ± 25	< 0.001
*High-sensitivity C‑reactive protein (mg/L)*	1.1 [0.5–2.3]	2.75 [1–4.45]	< 0.001 §
*Pro-brain natriuretic peptide (ng/L)*	97 [66–155]	885 [613–1447]	< 0.001 §
*Smoking categories (%)*			0.302
Never	1781 (41.9)	13 (33.3)	–
Former	1665 (39.1)	20 (51.3)	–
Current	809 (19.0)	6 (15.4)	–
*Alcohol drinkers (%)*	3469 (75.8)	38 (90.5)	0.027
*Alcohol consumption (units/week)*	3 [0–8]	7.5 [2.5–15.5]	0.001 §
*Physical activity (%)*	1280 (43.8)	2 (10.0)	0.002
*Risk of sleep apnea (%)*	708 (22.3)	8 (28.6)	0.426

Results are expressed as number of participants (percentage) for categorical data, as average ± standard deviation or as median [interquartile range] for continuous variables. Between-group comparisons using chi-square for categorical variables and Student’s *t *test or Kruskal–Wallis test (§) for continuous variables

*AF* atrial fibrillation, *FPG* fasting plasma glucose

Similar findings were obtained when comparing participants with and without AF + AFL (combined outcome; Table 2) or when restricting the analysis to participants with all covariates, except that no difference was found regarding dyslipidemia (Supplementary Table 3).

**Table 2:** Characteristics of participants with and without AF + AFL (combined outcome) at second follow-up, CoLaus|PsyCoLaus study, Lausanne, Switzerland

	Non-AF/AFL cases	AF + AFL cases	*p*
*N*	4564	52	
*Age (years)*	62 ± 10	74 ± 8	< 0.001
*Women (%)*	2525 (55.3)	14 (26.9)	< 0.001
*History of cardiovascular disease (%)*			
Personal	236 (5.2)	12 (23.1)	< 0.001
Family	2259 (49.5)	22 (42.3)	0.303
*Hypertension (%)*	2062 (46.5)	44 (84.6)	< 0.001
*Systolic BP (mm* *Hg)*	127 ± 18	129 ± 24	0.285
*Diastolic BP (mm* *Hg)*	77 ± 11	80 ± 13	0.041
*Body mass index (kg/m*^*2*^*)*	26.4 ± 4.7	28.8 ± 4.6	< 0.001
*Body mass index categories (%)*	–	–	0.001
Normal + underweight	1796 (41.7)	12 (23.1)	–
Overweight	1717 (39.8)	21 (40.4)	–
Obese	799 (18.5)	19 (36.5)	–
*Dyslipidemia (%)*	2043 (44.8)	31 (59.6)	0.032
*Diabetes using FPG (%)*	437 (10.1)	13 (25.0)	< 0.001
*Diabetes using HbA1c (%)*	422 (9.8)	13 (25.0)	< 0.001
*Creatinine (μmol/L)*	79 ± 21	92 ± 23	< 0.001
*High-sensitivity C‑reactive protein (mg/L)*	1.1 [0.5–2.3]	2.8 [1–4.55]	< 0.001 §
*Pro-brain natriuretic peptide (ng/L)*	96 [66–154]	907 [587–1447]	< 0.001 §
*Smoking categories (%)*	–	–	0.431
Never	1775 (41.8)	19 (40.4)	–
Former	1663 (39.2)	22 (46.8)	–
Current	809 (19.0)	6 (12.8)	–
*Alcohol drinkers (%)*	3461 (75.8)	46 (88.5)	0.034
*Alcohol consumption (units/week)*	3 [0–8]	6 [2–13]	0.009 §
*Physical activity (%)*	1277 (43.8)	5 (17.9)	0.006
*Risk of sleep apnea (%)*	707 (22.3)	9 (27.3)	0.493

Results are expressed as number of participants (percentage) for categorical data, as average ± standard deviation or as median [interquartile range] for continuous variables. Between-group comparisons using chi-square for categorical variables and Student’s *t* test or Kruskal–Wallis test (§) for continuous variables

*AF* atrial fibrillation, *AFL* atrial flutter, *FPG* fasting plasma glucose

Multivariable analysis identified increasing age and male gender as being positively associated with AF and AF + AFL (Table 3). The others risk factors associated with AF and AF + AFL also tended to be positively associated, but were not statistically significant.

**Table 3:** Multivariable analysis of the factors associated with AF or with combined AF + AFL at second follow-up, CoLaus|PsyCoLaus study, Lausanne, Switzerland

	AF	P‑value	AF + AFL	*p*
*Age (per decade)*	2.86 (1.40–5.87)	0.004	3.44 (1.78–6.67)	< 0.001
*Men vs. women*	4.98 (1.01–24.6)	0.049	7.47 (1.56–35.7)	0.012
*Personal history of CVD (yes vs. no)*	2.60 (0.56–12.0)	0.222	2.13 (0.48–9.43)	0.318
*Hypertension (yes vs. no)*	2.86 (0.58–14.1)	0.195	1.58 (0.46–5.41)	0.465
*Body mass index categories*				
Normal + underweight	1 (ref.)	–	1 (ref.)	–
Overweight	5.02 (0.60–42.1)	0.137	3.31 (0.69–16.0)	0.136
Obese	6.34 (0.64–62.4)	0.113	3.72 (0.60–23.0)	0.158
*Dyslipidemia (yes vs. no)*	0.45 (0.12–1.72)	0.242	0.41 (0.12–1.42)	0.161
*Diabetes using HbA*_*1*_*c (yes vs. no)*	0.91 (0.20–4.13)	0.901	1.35 (0.35–5.26)	0.668
*Creatinine (per 10* *μmol/L increase)*	1.04 (0.93–1.15)	0.513	1.02 (0.92–1.13)	0.715
*hs-CRP (per 1* *mg/L increase)*	1.10 (0.96–1.27)	0.166	1.10 (0.97–1.24)	0.155
*Alcohol drinkers (yes vs. no)*	1.67 (0.32–8.80)	0.542	1.14 (0.30–4.39)	0.849
*Physical activity (yes vs. no)*	0.88 (0.17–4.56)	0.884	1.66 (0.46–6.03)	0.439

Results are expressed as multivariable-adjusted odds ratio and 95% confidence interval (CI). Analysis performed with 2421 participants

*AF* atrial fibrillation, *AFL* atrial flutter, *CVD* cardiovascular disease, *hs-CRP* high-sensitivity C‑reactive protein

In the subgroup of participants with OSA data, multivariate analysis including the Berlin Questionnaire showed overall similar results, except that a high risk of OSA was not statistically significantly associated with AF or AF/AFL (Supplementary Table 4).

## Discussion

Our study reported an AF point prevalence of 0.9% (95% CI: 0.7–1.2%). This finding is similar to the population-based survey conducted in Geneva, Switzerland, using data from 2000, where the prevalence of AF among subjects aged ≥ 50 years was 0.9% [8]. Later, in the 2010s, the Swiss AF prevalence was estimated between 0.6% and 0.7% [12]. Therefore, our study shows an overall stability in the AF point prevalence in Lausanne, Switzerland. Whether the Swiss prevalence of AF will steeply increase in the future is uncertain and further studies are needed to explore prevalence of AF overtime.

The point prevalence of AF was higher among men, which is in agreement with previous studies [8, 12]. In the CoLaus|PsyCoLaus cohort, men had a more unfavorable cardiovascular risk factor profile, which can explain why they were at more risk of developing AF [14, 21, 22]. Moreover, according to the BiomarCaRE Consortium study, increased height seems to also be a risk factor in AF [23]. The larger cardiac dimension and higher excitability of the conduction system might also lead to a greater susceptibility to arrhythmia [24], but the exact pathophysiology must be studied further. On the other hand, a cohort study showed that, after adjusting for height and other AF risk factors, male sex was no longer significantly associated with AF [25, 26]. Indeed, the lifetime risk of developing AF is approximately 40% in women and men, but men develop it earlier with an incidence that increases rapidly after 50 years of age [12], whereas in women, the incidence increases a decade later [23, 24]. This phenomenon might be explained by the protector effect of estrogens [27–29].

Increasing age is one of the leading risk factors of AF [12]. In participants aged ≥ 80, the point prevalence of AF in our study peaked at 3.1% (95% CI: 2.0–4.2%). This finding confirms previously published results [5, 8, 30, 31]. As the human body ages, the arterial walls stiffen leading to atrial volume overload, which causes AF [1, 32]. Another reason is the presence of other cardiac comorbidities such as coronary artery disease and heart failure, which also increase with age [32].

Hypertension, higher BMI, personal history of CVD, diabetes, dyslipidemia, and physical activity were associated with AF in the bivariate analysis, which was expected as they are known risk factors for AF [1, 12, 32, 33]. Higher creatinine and pro-BNP levels were also associated with AF. However, these associations were no longer significant in the multivariable analysis. Our sample size for the multivariable analysis was smaller than for the bivariate analysis (*n* = 2421 participants) and therefore could explain the reduced statistical power. The trends of the OR still confirm the results previously published [1, 12, 32, 33]. Furthermore, according to the European Society of Cardiology guidelines, the accumulation of these comorbidities plays a major role in the development of AF [12]. Thus, early intervention and better control of modifiable risk factors should decrease the incidence of AF, but further studies are needed to confirm this.

No association was found between alcohol drinking and AF. An explanation is the relatively low level of alcohol consumption in our study (median of 7.5 units/week among AF cases), as AF is mostly associated with high levels of alcohol consumption (> 21 units/week; [34]).

No association was found between OSA and AF. For a sizable number of participants there were no data for the Berlin Questionnaire and the statistical power was therefore reduced. The link between OSA and AF should be examined better, using a larger number of patients with polysomnography data.

For AF + AFL, the point prevalence was 1.1% (95% CI: 0.4–1.5%) in our study, which is in line with previously published reports [8, 12]. The evolution is also stable along the years and further studies are needed to monitor the prevalence. The point prevalence of AF + AFL was also higher in men than in women. Increased height and larger cardiac dimension may also be responsible [23, 24]. The point prevalence of AF + AFL also peaked at 4.7% (95% CI: 3.4–6.0%) for participants aged ≥ 80, which was expected. Arterial stiffness and atrial volume overload may be also responsible. As with AF, the point prevalence of AF + AFL increased with the presence of modifiable risk factors, such as hypertension, higher BMI, heart failure, diabetes, sedentary lifestyle, and chronic kidney disease. However, the results of multivariable analysis were not significant, but the trends of the OR were similar to those with AF. Alcohol consumption and OSA were not associated AF + AFL.

### Study limitations

This study has some limitations. First, our study potentially excludes participants with more comorbidities, since participation in population-based studies are selective and healthy people participate more frequently [20]. Thus, our study might have underestimated the prevalence of AF and AFL. However, this effect could be partly counterbalanced by the fact that our study included only participants of Caucasian origin (inclusion criteria at baseline) and it is known that individuals of non-European ancestry have a lower prevalence of AF [35]. Furthermore, the prevalence of AF and AFL was derived from a single time point ECG, and therefore we could only calculate point prevalence, which means we might have underestimated the true AF prevalence, as paroxysmal AF might have been missed. Furthermore, we did not detect atypical AFL in our study, which is also caused by atrial volume overload and we cannot make a statement about its prevalence. Smartphone-based ambulatory monitoring will probably be an attractive alternative in the future [36]. Finally, the small number of participants with AF reduced the statistical power and precluded the identification of some determinants of AF.

## Conclusion

The prevalence of atrial fibrillation and of atrial fibrillation + atrial flutter, assessed between 2014 and 2017 in the city of Lausanne (Switzerland), was low but increased with age and in male participants.

## Supplementary Information

Supplementary Table 1: characteristics of included and excluded (without ECG) participants at 2nd follow-up, CoLaus|PsyCoLaus study, Lausanne, Switzerland

Supplementary Table 2: characteristics of included and excluded (without ECG or missing covariate(s) ≥ 1) participants at 2nd follow-up, CoLaus|PsyCoLaus study, Lausanne, Switzerland

Supplementary Table 3: characteristics of participants with and without AF, CoLaus|PsyCoLaus study, Lausanne, Switzerland. Analysis restricted to participants with all covariates

Supplementary table 4: multivariable analysis of the factors associated with AF or with AF + AFL at 2nd follow-up, CoLaus|PsyCoLaus study, Lausanne, Switzerland. Analysis including risk of sleep apnea

## Declarations

In accordance with the 1964 Helsinki Declaration and its later amendments. All participants signed the informed consent before any data was collected. The CoLaus|PsyCoLaus study was approved by the Ethic committee of the canton of Vaud (reference 16/03, reference 33/09, reference 26/14). This study reused the data collected for CoLaus|PsyCoLaus and its aim is within the initial objectives of the CoLaus|PsyCoLaus study. Therefore, no new approval from the Ethic committee of the canton of Vaud was needed. The principal investigators of the CoLaus|PsyCoLaus study were consulted and agreed to this study.
