# Supplementary material for: Prevalence of atrial fibrillation: The Swiss population-based CoLaus|PsyCoLaus study
Source: Herz. 2021 Dec 13;48(1):48–54. doi: 10.1007/s00059-021-05090-7 (PMC9892084; doi:10.1007/s00059-021-05090-7)
Supplement: Supplementary file 1 — Supplementary Table 1: characteristics of included and excluded (without ECG) participants at 2nd follow-up, CoLaus|PsyCoLaus study, Lausanne, Switzerland [file 59_2021_5090_MOESM1_ESM.pdf]

Prevalence of atrial fibrillation

The Swiss population-based CoLaus study

Herz

Daryoush Samim, MD [1]; Damien Choffat[1*] ; Peter Vollenweider, MD [1]; Gérard Waeber, MD [1]; Pedro Marques-Vidal, MD, PhD, FESC [1]; Marie Méan, MD [1]

[1] Department of medicine, internal medicine, Lausanne University Hospital and University of Lausanne, Switzerland.

The authors take responsibility for all aspects of the reliability and freedom from bias of the data presented and their discussed interpretation.

***Corresponding author (DC) :** Damien.choffat@chuv.ch

Supplementary Table 1: characteristics of included and excluded (without ECG) participants at 2nd follow-up, CoLaus|PsyCoLaus study, Lausanne, Switzerland

	Included	Excluded	P-value
N	4616	265	
Age (years)	62.5±10.2	70.6±10.9	<0.001
Woman (%)	2539 (55.0)	150 (56.6)	0.611
History of cardiovascular disease (%)			
Personal	248 (5.4)	42 (15.8)	<0.001
Family	2281 (49.4)	118 (44.5)	0.122
Hypertension (%)	2106 (47.0)	157 (78.1)	<0.001
Systolic BP (mmHg)	127±18	134±20	<0.001
Diastolic BP (mmHg)	77±11	76±11	0.294
Body mass index (kg/m^2)	26.5±4.7	26.9±5.2	0.236
Body mass index categories (%)			0.062
Normal + underweight	1808 (41.4)	49 (41.9)	
Overweight	1738 (39.8)	37 (31.6)	
Obese	818 (18.7)	31 (26.5)	
Dyslipidemia (%)	2074 (44.9)	142 (53.6)	0.006
Diabetes using FPG (%)	450 (10.3)	48 (27.9)	<0.001
Diabetes using HbA_1c (%)	435 (9.9)	47 (26.9)	<0.001
Creatinine (µmol/L)	79±21	85±29	<0.001
High-sensitivity C-reactive protein (mg/L)	1.1 [0.5 – 2.3]	1.9 [0.8 – 4]	<0.001 §
Pro-Brain natriuretic peptide (ng/L)	98 [66 - 160]	198 [84 - 499]	<0.001 §
Smoking categories (%)			0.367
Never	1794 (41.8)	92 (45.8)	
Former	1685 (39.2)	69 (34.3)	
Current	815 (19.0)	40 (19.9)	
Alcohol drinkers (%)	3507 (76.0)	199 (75.1)	0.744
Alcohol consumption (units/week)	3 [0 - 8]	2 [0 - 7]	<0.005 §
Physically active (%)	1282 (43.5)	5 (22.7)	0.050

Results are expressed as number of participants (percentage) for categorical data, as average ± standard deviation or as median [interquartile range] for continuous variables. Between-group comparisons using chi-square for categorical variables and student's test or Kruskal-Wallis test (§) for continuous variables.
