# Supplementary material for: Prevalence of atrial fibrillation: The Swiss population-based CoLaus|PsyCoLaus study
Source: Herz. 2021 Dec 13;48(1):48–54. doi: 10.1007/s00059-021-05090-7 (PMC9892084; doi:10.1007/s00059-021-05090-7)
Supplement: Supplementary file 2 — Supplementary Table 2: characteristics of included and excluded (without ECG or missing covariate(s) ≥ 1) participants at 2nd follow-up, CoLaus|PsyCoLaus study, Lausanne, Switzerland [file 59_2021_5090_MOESM2_ESM.pdf]

Prevalence of atrial fibrillation

The Swiss population-based CoLaus study

Herz

Daryoush Samim, MD [1]; Damien Choffat[1*] ; Peter Vollenweider, MD [1]; Gérard Waeber, MD [1]; Pedro Marques-Vidal, MD, PhD, FESC [1]; Marie Méan, MD [1]

[1] Department of medicine, internal medicine, Lausanne University Hospital and University of Lausanne, Switzerland.

The authors take responsibility for all aspects of the reliability and freedom from bias of the data presented and their discussed interpretation.

***Corresponding author (DC) :** Damien.choffat@chuv.ch

Supplementary Table 2: characteristics of included and excluded (without ECG or missing covariate(s) ≥ 1) participants at 2nd follow-up, CoLaus|PsyCoLaus study, Lausanne, Switzerland

	Included	Excluded	P-value
N	3539	1342	
Age (years)	62.1±10.0	65.1±11.2	<0.001
Woman (%)	1943 (54.9)	746 (55.6)	0.667
History of cardiovascular disease (%)			
Personal	174 (4.9)	116 (8.6)	<0.001
Family	1757 (49.6)	642 (47.8)	0.259
Hypertension (%)	1602 (45.3)	661 (57.6)	<0.001
Systolic BP (mmHg)	127±18	128±19	0.006
Diastolic BP (mmHg)	78±11	77±11	0.163
Body mass index (kg/m^2)	26.5±4.6	26.2±5.1	0.107
Body mass index categories (%)			0.002
Normal + underweight	1427 (40.3)	430 (45.6)	
Overweight	1448 (40.9)	327 (34.7)	
Obese	664 (18.8)	185 (19.6)	
Dyslipidemia (%)	1642 (46.4)	574 (42.8)	0.023
Diabetes using FPG (%)	324 (9.2)	174 (17.2)	<0.001
Diabetes using HbA_1c (%)	311 (8.8)	171 (16.8)	<0.001
Creatinine (µmol/L)	79±20	79±26	0.719
High-sensitivity C-reactive protein (mg/L)	1.1 [0.5 – 2.3]	1.4 [0.6 – 2.9]	<0.001 §
Pro-Brain natriuretic peptide (ng/L)	96 [65 - 154]	120 [72 - 225]	<0.001 §
Smoking categories (%)			0.696
Never	1489 (42.1)	397 (41.5)	
Former	1386 (39.2)	368 (38.5)	
Current	664 (18.8)	191 (20.0)	
Alcohol drinkers (%)	2614 (73.9)	1092 (81.4)	<0.001
Alcohol consumption (units/week)	4 [0 - 8]	3 [0 - 7]	<0.001 §
Physically active (%)	1044 (40.1)	243 (44.7)	0.501

Results are expressed as number of participants (percentage) for categorical data, as average ± standard deviation or as median [interquartile range] for continuous variables. Between-group comparisons using chi-square for categorical variables and student’s test or Kruskal-Wallis test (§) for continuous variables.
