# Supplementary material for: Prevalence of atrial fibrillation: The Swiss population-based CoLaus|PsyCoLaus study
Source: Herz. 2021 Dec 13;48(1):48–54. doi: 10.1007/s00059-021-05090-7 (PMC9892084; doi:10.1007/s00059-021-05090-7)
Supplement: Supplementary file 3 — Supplementary Table 3: characteristics of participants with and without AF, CoLaus|PsyCoLaus study, Lausanne, Switzerland. Analysis restricted to participants with all covariates [file 59_2021_5090_MOESM3_ESM.pdf]

Prevalence of atrial fibrillation

The Swiss population-based CoLaus study

Herz

Daryoush Samim, MD [1]; Damien Choffat[1*] ; Peter Vollenweider, MD [1]; Gérard Waeber, MD [1]; Pedro Marques-Vidal, MD, PhD, FESC [1]; Marie Méan, MD [1]

[1] Department of medicine, internal medicine, Lausanne University Hospital and University of Lausanne, Switzerland.

The authors take responsibility for all aspects of the reliability and freedom from bias of the data presented and their discussed interpretation.

***Corresponding author (DC) :** Damien.choffat@chuv.ch

Supplementary Table 3: characteristics of participants with and without atrial fibrillation, CoLaus|PsyCoLaus study, Lausanne, Switzerland. Analysis restricted to participants with all covariates.

	**Non-AF cases**	**AF cases**	**P-value**
N	3505	34	
Age (years)	62±10	74±7	<0.001
Woman (%)	1937 (55.3)	6 (17.6)	<0.001
History of cardiovascular disease (%)			
Personal	163 (4.7)	11 (32.4)	<0.001
Family	1743 (49.7)	14 (41.2)	0.321
Hypertension (%)	1571 (44.8)	31 (91.2)	<0.001
Systolic BP (mmHg)	126±17	130±24	0.213
Diastolic BP (mmHg)	77±10	80±15	0.137
Body mass index (kg/m^2)	26.5±4.6	29.3±4.8	<0.001
Body mass index categories (%)			0.001
Normal + underweight	1421 (40.5)	6 (17.6)	
Overweight	1434 (40.9)	14 (41.2)	
Obese	650 (18.5)	14 (41.2)	
Dyslipidemia (%)	1622 (46.3)	20 (58.8)	0.144
Diabetes using FPG (%)	316 (9.0)	8 (23.5)	0.003
Diabetes using HbA1c (%)	302 (8.6)	9 (26.5)	<0.001
Creatinine (µmol/L)	79±20	95±26	<0.001
hs-CRP (mg/L)	1.1 [0.5 – 2.2]	2.7 [0.8 – 4.4]	0.001 §
Pro-BNP (ng/L)	95 [65 - 150]	913 [587 - 1615]	<0.001 §
Smoking categories (%)			0.255
Never	1478 (42.2)	11 (32.4)	
Former	1368 (39.0)	18 (52.9)	
Current	659 (18.8)	5 (14.7)	
Alcohol drinkers (%)	2583 (73.7)	31 (91.2)	0.021
Alcohol consumption (units/week)	4 [0 - 8]	8 [3 – 17]	<0.001 §
Physical activity (%)	1042 (43.3)	2 (14.3)	0.029

BP, blood pressure; FPG, fasting plasma glucose; hs-CRP, high-sensitivity c-reactive protein; pro-BNP, pro-Brain natriuretic peptide. Results are expressed as number of participants (percentage) for categorical data, as average ± standard deviation or as median [interquartile range] for continuous variables. Between-group comparisons using chi-square for categorical variables and student's test or Kruskal-Wallis test (§) for continuous variables
