# Supplementary material for: Prevalence of atrial fibrillation: The Swiss population-based CoLaus|PsyCoLaus study
Source: Herz. 2021 Dec 13;48(1):48–54. doi: 10.1007/s00059-021-05090-7 (PMC9892084; doi:10.1007/s00059-021-05090-7)
Supplement: Supplementary file 4 — Supplementary table 4: multivariable analysis of the factors associated with AF or with AF + AFL at 2nd follow-up, CoLaus|PsyCoLaus study, Lausanne, Switzerland. Analysis including risk of sleep apnea [file 59_2021_5090_MOESM4_ESM.pdf]

Prevalence of atrial fibrillation

The Swiss population-based CoLaus study

Herz

Daryoush Samim, MD [1]; Damien Choffat[1*] ; Peter Vollenweider, MD [1]; Gérard Waeber, MD [1]; Pedro Marques-Vidal, MD, PhD, FESC [1]; Marie Méan, MD [1]

[1] Department of medicine, internal medicine, Lausanne University Hospital and University of Lausanne, Switzerland.

The authors take responsibility for all aspects of the reliability and freedom from bias of the data presented and their discussed interpretation.

***Corresponding author (DC) :** Damien.choffat@chuv.ch

Supplementary table 4: multivariable analysis of the factors associated with atrial fibrillation or with combined atrial fibrillation + atrial flutter at 2nd follow-up, CoLaus|PsyCoLaus study, Lausanne, Switzerland. Analysis including risk of sleep apnea.

	**AF**	**P-value**	**AF + AFL**	**P-value**
Age (per decade)	2.41 (1.08 - 5.38)	0.033	2.70 (1.29 - 5.66)	0.009
Man vs. woman	8.25 (0.98 - 69.4)	0.052	10.7 (1.30 - 87.4)	0.028
Personal history of CVD (yes vs. no)	3.44 (0.53 - 22.4)	0.195	2.92 (0.48 - 17.6)	0.243
Hypertension (yes vs. no)	3.30 (0.63 - 17.3)	0.157	2.34 (0.57 - 9.62)	0.240
Body mass index categories				
Normal + underweight	1 (ref.)		1 (ref.)	
Overweight	3.78 (0.43 - 32.9)	0.229	4.98 (0.60 - 41.6)	0.139
Obese	7.19 (0.68 - 75.8)	0.101	6.88 (0.66 - 72.1)	0.108
Dyslipidemia (yes vs. no)	0.29 (0.06 - 1.39)	0.123	0.30 (0.07 - 1.24)	0.096
Diabetes using HbA_1c (yes vs. no)	1.89 (0.37 - 9.61)	0.444	2.58 (0.60 - 11.0)	0.201
Creatinine (per 10 µmol/L increase)	1.07 (0.97 - 1.19)	0.172	1.05 (0.95 - 1.16)	0.334
hs-CRP (per 1 mg/L increase)	1.13 (0.95 - 1.34)	0.161	1.13 (0.97 - 1.32)	0.110
Alcohol drinkers (yes vs. no)	1.12 (0.21 - 5.98)	0.892	0.76 (0.19 - 3.03)	0.700
Physical activity (yes vs. no)	1.12 (0.02 - 6.32)	0.895	1.66 (0.37 - 7.45)	0.505
Risk of sleep apnea (high vs. low)	0.25 (0.05 - 1.34)	0.105	0.41 (0.10 - 1.66)	0.209

AF, atrial fibrillation; AFL, atrial flutter; CVD, cardiovascular disease. hs-CRP, high-sensitivity C-reactive protein. Results are expressed as multivariable-adjusted odds ratio and 95% confidence interval (CI). Analysis performed on 1920 participants.
